# Surgical Management of Foot Syndactyly: The State of the Art and a Treatment Algorithm Based on a Literature Review

**DOI:** 10.3390/jcm14030830

**Published:** 2025-01-27

**Authors:** Elena Artioli, Alessandro Cargeli, Antonio Mazzotti, Simone Ottavio Zielli, Alberto Arceri, Laura Langone, Federico Sgubbi, Gianmarco Di Paola, Cesare Faldini

**Affiliations:** 1 1st Orthopaedics and Traumatologic Clinic, IRCCS Istituto Ortopedico Rizzoli, 40136 Bologna, Italyalessandro.cargeli@ior.it (A.C.); simoneottavio.zielli@ior.it (S.O.Z.); alberto.arceri@ior.it (A.A.); laura.langone@ior.it (L.L.); federico.sgubbi@ior.it (F.S.); gianmarco.dipaola@ior.it (G.D.P.);; 2 Department of Biomedical and Neuromotor Sciences (DIBINEM), Alma Mater Studiorum University of Bologna, 40123 Bologna, Italy

**Keywords:** syndactyly, foot, algorithm, surgical treatment

## Abstract

**Objectives:** This systematic review provides an in-depth analysis of the surgical treatment of foot syndactyly, offering insights into the various options and their applications. The objective is to improve treatment standardization and optimize patient care in cases of foot syndactyly. **Methods:** By synthesizing the existing evidence, the authors propose a treatment algorithm to guide clinicians in decision-making, considering the type and severity of syndactyly. **Results**: Sixteen articles were included in the systematic review, comprising a total of 395 patients. The mean age at surgery was 6 years (range: 0.66–23 years). Clinical outcomes were assessed after a mean follow-up of 33.2 months (range: 4–82.8 months). Several surgical procedures were described in the included articles. **Conclusions**: Continued research efforts and collaborative initiatives are crucial to further refine our understanding of complications and enhance surgical practices in foot syndactyly procedures.

## 1. Introduction

Syndactyly (Greek Syn = together; Dactylos = digit) is a digital malformation in which adjacent fingers and/or toes are webbed because they fail to separate during limb development. It is a congenital anomaly that affects a significant proportion of the global population [1].

The incidence in the foot has been reported to be 1:1000 to 1:3000, with a predilection for males over females of 3:1 [1,2]. Persistent webbing in the foot most often occurs in the second interspace [3,4]. The incidence of bilateral foot involvement ranges between 35 and 50% [1,3,5]. A positive family history may be present in 10 to 40% of cases [6].

Syndactyly occurs when apoptosis or programmed cell death during gestation is absent or incomplete [2]. Webbed toes occur most commonly in familial syndactyly and Down syndrome. It is also associated with a number of rare conditions, notably, Apert syndrome, Klippel–Fleil syndrome, Carpenter syndrome, Bardet–Biedl syndrome, Miller syndrome, Smith–Lemli–Opitz syndrome, Timothy syndrome, and ectodermal dysplasia [1].

It is important to determine the extent of syndactylization, especially in the presence of osseous union. In 1932, Kanavel first classified syndactylism as it related to the hand into degrees based on the severity of the fusion [7]. In first-degree syndactyly, only the skin and subcutaneous tissues are involved, whereas second-degree syndactyly indicates an element of osseous union. First- and second-degree syndactylization are amenable to surgical correction. The third and fourth degrees indicate a severe alteration of the anatomy that obviates surgical intervention on the premise that a restoration of normal function cannot be expected [6,8].

While syndactyly often poses minimal functional limitations, certain cases may lead to significant discomfort, impaired mobility, and cosmetic considerations with psychological distress for affected individuals [7,8].

The clinical presentation of syndactyly of the foot can include:

- Fused toes—The most obvious sign is the fusion of two or more toes. The extent of fusion can range from partial to complete.
- Webbing—The skin and soft tissues between the fused toes may form a web-like structure.
- Toe deformities—In some cases, there may be associated toe deformities, such as the absence of certain toe bones or an abnormal alignment.
- Limited toe movement—Depending on the degree of fusion, there may be limited movement of the affected toes [8,9].

Syndactyly can be addressed through both non-surgical and surgical approaches [10,11]. In cases of uncomplicated toe syndactyly where there is no impact on function, non-surgical management is typically preferred. Surgical intervention, in these instances, primarily serves cosmetic purposes and should only be considered after a comprehensive discussion of the potential risks with the patient and their family [10,11]. Non-surgical management is also indicated for cases of partial syndactyly and webbing between the toes associated with supernumerary digits [12,13].

Toe syndactyly separation has emerged as an effective intervention to alleviate functional and cosmetic issues related to fused toes [10,13]. The specific surgical technique used can vary based on the severity of the syndactyly, the number of toes involved, and individual patient factors.

While there are several well-established algorithms for the treatment of hand syndactyly [7], a standardized treatment algorithm for foot syndactyly surgery is lacking. The existing protocols are tailored to the unique functional needs of the hand, which differ significantly from the requirements of the foot. Therefore, the goal of this systematic review was to evaluate the existing literature on foot syndactyly separation and propose an effective treatment algorithm to guide clinical decision-making.

## 2. Materials and Methods

### 2.1. Search Strategy and Selection Criteria

This systematic review was conducted according to the PRISMA (Preferred Reported Items of Systematic Reviews and Meta-analyses) guidelines (the PRISMA checklist is provided in the Supplementary Materials). The review was not registered on PROSPERO. A systematic search was conducted across electronic databases, including PubMed, Embase, and Cochrane Library, to identify relevant studies published until 25 May 2024 on foot syndactyly separation (the surgical treatment of syndactyly) using a combination of the following keywords: syndactyly, complications, and foot. Additional articles were found through a cross-reference search of the eligible studies.

The inclusion criteria consisted of articles discussing the surgical treatment of syndactyly using the following parameters: papers written in English, original articles, and case reports focused on the surgical treatment of syndactyly in the foot, specifically those reporting data on the surgical techniques used and the postoperative course. In contrast, the exclusion criteria included articles concerning syndactyly of the hand, articles where the surgical technique was not described, articles without data on the postoperative course, and non-English articles.

After duplicate removal, two authors independently reviewed the titles and abstracts of all the articles to select those eligible for inclusion; in the case of a disagreement, the senior author made the final decision. The full texts of the retrieved studies were carefully examined to extract the following information:

1. Article data—authors, year, study type, level of evidence (LOE);
2. Population data—number of patients, number of webs, toes involved, sex of patients, mean age at surgery, mean follow-up;
3. Surgical procedure and indication;
4. Complications and their management.

### 2.2. Statistical Analysis

Categorical data are presented in terms of frequencies and/or percentages, while continuous variables are expressed as the mean values along with the ranges. Data collection was performed using Microsoft Excel (Microsoft Corporation, Redmond, WA, USA) for Windows 10.

## 3. Results

### 3.1. Study Selection

**Figure 1:**
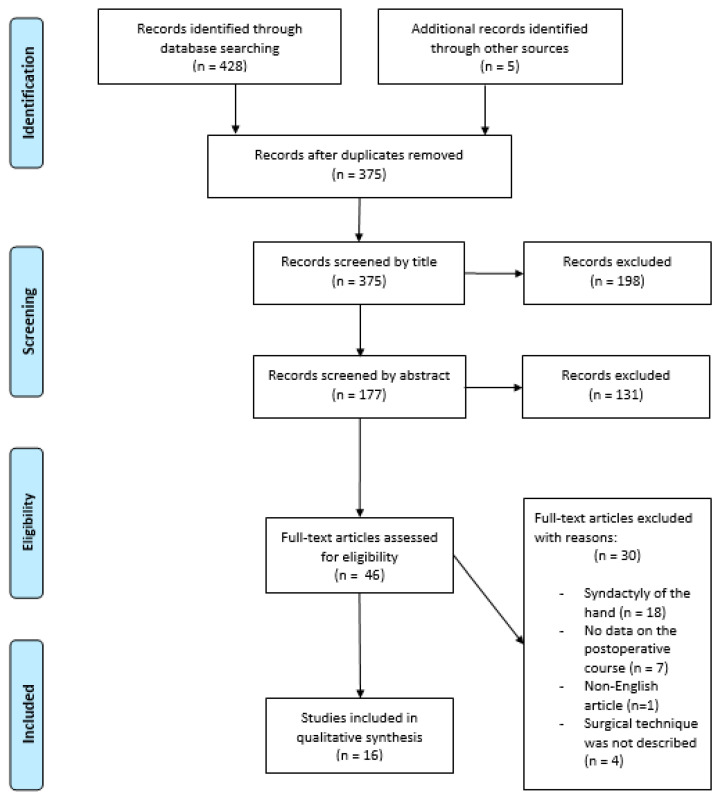
PRISMA flowchart showing the study selection process.

Following the database search, a total of 428 records were identified, with an additional 5 studies retrieved through manual cross-referencing. After the removal of duplicates, 375 unique articles remained for title and abstract screening. Subsequently, 46 full-text articles were assessed for eligibility, of which 16 met the predefined inclusion criteria and were incorporated into the systematic review. The study selection process is outlined in Figure 1.

Of the included articles, twelve (75%) were retrospective case series, classified as LOE IV according to the Oxford Level of Evidence scale, while the remaining four (25%) were case reports, classified as LOE V. The considered papers were published between 1995 and 2020, with the majority published in the last ten years (Table 1).

### 3.2. Population Data

The total number of included patients was 395. Only 11 articles reported the gender distribution: in total 74 females (48%) and 83 males (52%) were included. When reported, the mean age at surgery was 6 years (range 0.66–23 years). Patients were assessed for clinical outcomes after a mean follow-up of 33.2 months (range 4–82.8 months); only one study did not indicate the follow-up period [21].

Thirteen of the sixteen studies reported the interdigital space involved. The second interdigital space was the most commonly affected.

### 3.3. Risk of Bias Assessment of the Studies

The results of the risk of bias assessment of the included studies using the ROBINS-I tool are presented in Table 2.

### 3.4. Surgical Procedure and Indication

The surgical procedures [28] used in the included studies for the treatment of foot syndactyly were as follows:

1. Simple division (web space release)—The authors divided the soft tissue between the fused toes, creating separate toes. A zig-zag or Z-shaped incision (to avoid straight-line scarring) was often used to separate the fused skin between the digits. In cases of soft tissue syndactyly (without bone involvement), the skin was incised to promote the separation between the toes. This technique was adopted for cases with minimal soft tissue involvement [26,29].
2. Z-plasty—The authors made Z-shaped incisions to rearrange and lengthen the soft tissue, providing additional length to the web space. This technique was adopted for more complex cases where a straight-line incision may not be sufficient [17].
3. VM-plasty—One triangular flap was designed next to the web region on the dorsal site, whereas the remaining two triangular flaps, which were designed in an adjacent manner and called “M flap,” were placed on the volar site. Avoiding damage to the neurovascular bundles of the fingers on lateral sites is needed. The dorsal triangular flap was then placed between the volar adjacent triangular flaps [17].
4. Skin grafts—The authors used skin grafts to cover the raw areas where there was a shortage of skin after separation. This technique was adopted for cases with a deficiency of skin following separation [17,26,30,31].
5. Flap techniques—The authors used adjacent tissue flaps to cover the exposed areas after separation. This technique was adopted for cases with extensive soft tissue involvement [12,16,25].
6. Toe transposition—The authors transposed a toe to provide additional tissue for reconstruction in severe cases where there was a lack of adequate soft tissue. This technique was adopted for complex cases with a lack of tissue [25].
7. Osteotomy—The authors performed bone division with an osteotomy in addition to soft tissue separation. This technique was adopted for cases where bony fusion was present [32]. It should be considered in the treatment plan when there was significant osseous fusion. For non-bony syndactyly, the focus was primarily on soft tissue techniques such as Z-plasty, VM-plasty, and skin grafting.
8. Newer techniques—Dorsal triangular flaps and hyaluronic acid ester matrix were used in skin resurfacing.

### 3.5. Complications

This review identified a range of postoperative complications associated with foot syndactyly separation, including wound-related issues, scar complications, infections, and functional impairments. The incidence of these complications varied among the studies, as detailed in Table 3.

### 3.6. Treatment Algorithm Based on a Literature Review

Based on the findings from the literature review, an attempt was made to formulate a treatment algorithm tailored to the specific conditions of foot syndactyly.

**Figure 2:**
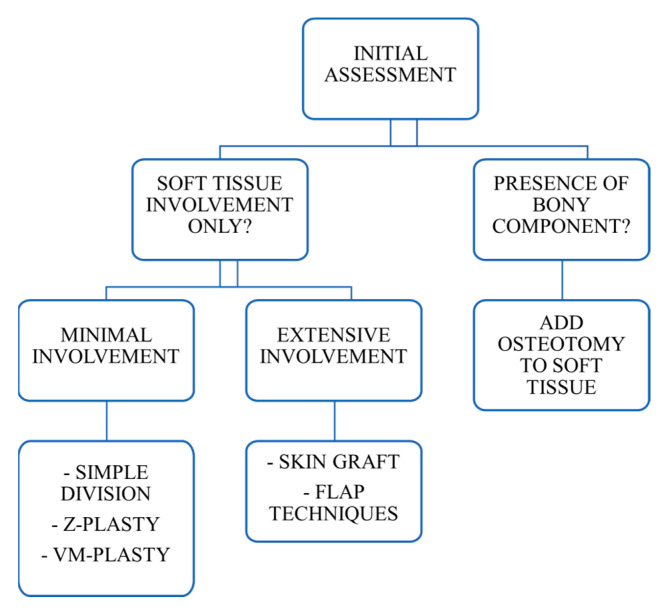
Treatment algorithm based on the condition of foot syndactyly.

For cases involving solely soft tissue, it is essential to distinguish between minimal and extensive involvement. In instances of minimal soft tissue involvement (situations where only a small area of soft tissue, like skin, subcutaneous tissue, etc., is affected or compromised), the therapeutic options encompass (1) simple division, (2) Z-plasty, and (3) VM plasty. Conversely, for extensive soft tissue involvement (deeper structures such as muscles or fascia), the recommended approaches include (1) skin grafts, (2) flap techniques, and (3) toe transposition. When a bony component is also present, osteotomy should be integrated into the treatment strategy. It is important to note that in certain cases, a combination of techniques may be applied to achieve optimal outcomes (Figure 2).

## 4. Discussion

The scientific literature on the surgical treatment of foot syndactyly is limited to small case series, underscoring the infrequency of this procedure. In this review, the surgical procedure for syndactyly was predominantly performed at a young age, as indicated by the mean age of the study population, which was 6 years, with an upper age limit of 23 years.

Surgery for foot syndactyly was considered when there were functional impairments, such as difficulties in walking and maintaining balance, or when there were cosmetic concerns. The decision to pursue surgery was often influenced by the severity of the syndactyly and its impact on the individual’s overall well-being [8].

Several surgical techniques have been employed. In the included studies, various methods for separating foot syndactyly and reconstructing web spaces were reported. Traditionally, a zig-zag incision was often used for separating the fingers to prevent postoperative contracture. Historically, toe syndactyly was also divided in a staggered fashion, often resulting in prominent scars on both the dorsal and plantar sides. Advances in surgical techniques have sought to reduce these visible scars [4,8].

The reconstruction of web spaces necessitates compensating for the dermal tissue deficiency that results from splitting the digital units. This review showed that this reconstruction was typically achieved through skin grafting, the use of local flaps, or a combination of both. While skin grafting is a straightforward and well-known method, it does not match the elasticity and texture provided by local flaps. Consequently, several modifications have been developed to avoid the need for skin grafts [4,28]. Tetsushi Aizawa et al. employed skin grafting for all surgeries, regardless of the completeness of syndactyly, favoring PSVN (preserved subcutaneous vascular network) skin grafts over traditional full-thickness or split-thickness skin grafts to reduce postoperative retraction rates [21].

Tsukada emphasized the importance of preserving the areolar and fat tissue immediately below the dermal layer to prevent the damage typically seen in full-thickness or split-thickness skin grafts. This intact dermal layer maintains the graft’s elasticity and texture. The preserved areolar tissue, with its well-developed vascular network, facilitates capillary anastomosis between the recipient site and the graft, minimizing postoperative degeneration. Additionally, the extracellular matrix of the areolar tissue, rich in collagen, reticular fibers, elastin fibers, and proteoglycans, serves as a barrier against fibrous adhesion to the wound bed [32].

Despite these advantages, skin grafting is often associated with pigmentation issues, particularly when grafts are harvested from unexposed areas such as the groin. While these regions can conceal donor site scars, they frequently exhibit significant pigmentation, especially in individuals with darker skin tones. As a result, Tetsushi Aizawa and his working group prioritized the quality of skin grafts over the visibility of donor site scars, preferring to harvest grafts from the ankle region [21]. Scars in this area are less noticeable in daily life, as the lateral sides of the digits are seldom exposed. To further minimize visible scarring, particularly on the dorsal surface of the foot, they adopted a linear incision technique for syndactyly dissection, in contrast to the traditional zig-zag approach. This method, combined with a small triangular dorsal flap, reduces skin defects and the risk of scar contracture, particularly when using PSVN skin grafts [21].

An alternative to skin grafts is flaps. However, reconstructing new web spaces using only flaps has shown limited success. Case studies of complete syndactyly treated exclusively with local flaps have reported postoperative contracture or keloid formation [16,26].

The VM-plasty technique, first introduced by Alexander et al. for post-burn syndactyly, was later adapted by Onishi et al. for toe syndactyly and scar contractures in other anatomical regions. VM-plasty is a relatively simple procedure that does not require skin grafting, making it accessible for less experienced surgeons [17,30,31]. Furthermore, Mericli et al. demonstrated the reliability of the tapered M-to-V flap in web space reconstruction, showing advantages over the conventional rectangular flap approach [25].

VM-plasty eliminates the need for skin grafts by using local flaps to reconstruct the web space [17,25]. Similar to Z-plasty [17], this method was shown to reduce donor site morbidity, minimize the risk of graft failure, and had a low recurrence rate. The resulting M-shaped scars are nonlinear, which decreases the likelihood of recurrence. Long-term outcomes with VM-plasty have shown no recurrence in reported cases. Additionally, the absence of skin grafts in VM-plasty simplifies postoperative care, allowing for early rehabilitation and reducing hospitalization times [17]. The procedure involved designing a triangular flap adjacent to the dorsal web region and two corresponding triangular flaps on the volar surface. These flaps were carefully elevated subcutaneously to avoid damage to the neurovascular bundles, resulting in effective and aesthetically favorable web space reconstruction [17,25].

In summary, although traditional methods such as zig-zag incisions and skin grafting remain effective, VM-plasty offers significant advantages [32]. This technique has been shown to simplify both the surgical process and postoperative care, delivering superior aesthetic and functional outcomes for patients undergoing syndactyly correction. Continued research and clinical experience will likely refine these techniques, further improving their safety and efficacy [32].

In cases of extensive syndactyly, skin grafting may still be necessary [33].

More recently, several techniques that eliminated the need for skin grafts—such as open treatment, primary closure by defatting [7,8], and intricate local flaps [9,10]—have been reported. These approaches are advantageous in that they reduce the operation time and donor site morbidity. However, they are associated with increased risks, such as sensory deficits from excessive subcutaneous fat removal, wound dehiscence due to tension, and secondary complications, including web creep, flexion contracture, and angulation deformity.

The use of split-thickness skin grafts at defect sites can result in angulation deformity or flexion contracture [11], and grafts harvested from penile skin may cause hyperpigmentation and flexion contracture [12]. Furthermore, open treatment poses a higher risk of adhesion at the division site.

Alternatively, some authors have advocated for using various flap techniques. Yi-Jia Lim introduced a novel technique employing a dorsal pentagonal island flap, which eliminates the need for skin grafting. This single-stage method combines a dorsal pentagonal island flap with dorsal and plantar triangular flaps for direct closure. The key advantages of this technique included reduced morbidity due to the avoidance of skin grafting and a shorter surgical duration [20]. However, this method is not without complications. Reported issues include partial synechiae, cellulitis, and keloid formation [16]. Despite these potential complications, the dorsal pentagonal island flap remains a viable option for single-stage web space reconstruction in both syndactyly and polysyndactyly cases, often yielding satisfactory functional and cosmetic outcomes [20].

Various flap techniques have been employed in syndactyly repair, depending on the extent of the deformity and the patient’s specific needs. These techniques include Z-plasty, dorsal flaps, V-Y advancement flaps, H-flaps, the four-flap technique, local random pattern flaps, and cross-finger flaps [11,16,25]. In some cases, skin grafts may be combined with flaps to ensure adequate healing [28].

Juan Liu et al. introduced the hexagonal advancement flap as a novel approach for syndactyly repair, eliminating the need for skin grafts. Their technique involved designing a “plane-shaped” advancement flap for the hand or dorsal foot, enabling web reconstruction with primary closure. This approach consistently achieved satisfactory cosmetic and functional outcomes, with no need for subsequent revisions. The dorsal planar advancement flap, without the use of skin grafts, represents a viable solution for reconstructing web spaces in simple syndactyly, particularly in the foot [23].

Susumu Saito et al. focused on the aesthetic goals of syndactyly surgery and introduced a transversely oriented transposition flap for dorsal web reconstruction, minimizing longitudinal scars on the dorsal toes [9]. Their retrospective case series highlighted the need to reconstruct not only functional but also aesthetically pleasing digits. However, longitudinal scars on the dorsal surface may not always meet patient expectations, as they serve as a constant reminder of the surgical intervention [9]. To address these concerns, they employed a transversely oriented transposition flap, which allows for maximal preservation of the dorsal interdigital skin and minimizes donor site morbidity due to the favorable healing potential of transverse scars. This technique was presented as a viable alternative for web reconstruction in toe syndactyly, particularly when achieving a high cosmetic outcome was a priority [9].

Despite advancements in flap techniques, some cases still necessitate the combined use of flaps and skin grafts. Jong Ho Kim et al. conducted a retrospective clinical study involving 118 patients over 25 years. Surgeries were performed using a dorsal triangular flap and a full-thickness skin graft [26].

Finally, meticulous postoperative care, including wound management, immobilization, and physical therapy, is crucial for optimal healing and functional outcomes following syndactyly surgery. Strict adherence to these protocols is essential for preventing complications such as infection, wound dehiscence, hypertrophic scarring, skin graft sloughing, and recurrence of the deformity [9,22,28]. Although complications can occur despite optimal care, some techniques, such as the prolonged use of web space bandages, have been reported to produce finer scars, as noted by Susumu Saito et al. [9]. Additionally, Kyung Rae Ko et al. addressed the issue of web creep following syndactyly surgery, emphasizing the importance of minimizing wound complications to achieve favorable cosmetic results. The application of compressive dressings combined with splinting can immobilize the web space and neighboring toes during the initial postoperative phase, thereby promoting optimal healing conditions. Moreover, employing sterile materials in conjunction with strict aseptic techniques can significantly reduce the incidence of early postoperative infections [22].

### 4.1. Limitations

This is the only systematic review addressing the surgical treatment of foot syndactyly that reports results from a comparable number of cases found in the literature. To obtain a significant number of cases, it was necessary to include case reports, despite their lower level of evidence. However, since 25% of the articles consisted of case reports, excluding them would likely have led to the loss of valuable information on surgical techniques that may be less commonly performed. Moreover, many studies included in this review were limited by small sample sizes, which can affect the generalizability of the results and increase the risk of bias.

Systematizing this topic was particularly challenging due to the lack of uniformity in clinical presentation, surgical treatment, and methods of outcome evaluation. The only potentially standardizable aspect was complications, but unfortunately, they were rarely described in detail. Moreover, the exclusion of non-English studies may have led to the omission of valuable data and introduced the potential for publication bias.

The use of various surgical techniques, each with numerous variants, made it difficult to develop a comprehensive treatment algorithm. The lack of uniform preoperative characteristics is a significant limitation, which stems from the fact that the pathology itself can present in diverse ways. The distinction between minimal or extensive involvement is unfortunately not clearly objective in the studies examined. Moreover, there are no standardized scoring systems to assess the outcomes of surgical procedures for foot syndactyly, and since this surgery often addresses the aesthetic impact of the condition, establishing objective standards is difficult.

The intention of this review was to propose a more detailed algorithm incorporating patient selection criteria, contraindications, and timing considerations. Unfortunately, the articles included in our review do not provide this information in a form that allows it to be synthesized into such an algorithm.

Therefore, the high variability in study designs, outcome measures, and data reporting made a meta-analysis unfeasible and potentially misleading due to inconsistencies and the lack of sufficient statistical data.

Consequently, it is challenging to draw definitive conclusions on this topic given the current literature, which is limited by a scarcity of high-quality studies, with only a few retrospective case series and the rest being case reports. Recognizing these limitations is essential for a comprehensive and accurate interpretation of the study’s results. Improved quality and greater uniformity of scientific articles are necessary to enable a truly systematic review of the surgical treatment of foot syndactyly.

### 4.2. Future Research Directions for Foot Syndactyly Surgery

Future research in foot syndactyly surgery should focus on several key areas to enhance outcomes. Long-term studies are needed to assess the durability of surgical results, including joint mobility and the recurrence of webbing, as well as the optimal timing for surgery. The development of standardized outcome measures for both aesthetic and functional results for quality of life, would improve consistency and inform clinical decision-making. Large-scale, multi-center studies incorporating randomized controlled trials are crucial for evaluating the effectiveness and cost-effectiveness of different surgical approaches.

Additionally, novel techniques such as 3D-printed scaffolds and tissue engineering [33] could address the limitations of traditional skin grafts and flaps, minimizing complications and improving results. Genetic research could also provide insights into the pathophysiology of syndactyly, enabling more personalized treatments and reducing recurrence. Advancements in tissue regeneration and biomaterials may further refine surgical methods, requiring less postoperative care and reducing complications.

In conclusion, these research areas are critical for improving surgical practices and patient outcomes for foot syndactyly.

## 5. Conclusions

This systematic review provides a comprehensive overview of the surgical treatment of foot syndactyly, offering insights into the various options and their applications. By synthesizing the existing evidence, this review aimed to guide clinicians in optimizing patient care and outcomes in reconstructive foot surgery.

The methodological differences between the studies reviewed significantly impact their comparability. Key differences in study design, patient populations, surgical techniques, postoperative management, and outcome reporting all contribute to the difficulty in drawing firm conclusions across the studies. The standardization of patient selection criteria, surgical approaches, and outcome reporting, as well as the inclusion of control groups and longer follow-up, would improve the quality of future studies in this field.

Continued research efforts and collaborative initiatives are crucial for further refining our understanding of complications and enhancing surgical practices for foot syndactyly procedures.

## Figures and Tables

**Table 1:** Summary of the included articles.

Author and Year	Type of Study	N° of Patients	N° of Feet/Webs	Females	Males	Mean Age at Surgery (Years)	Range/sd	Interdigital Spaces Involved	Surgical Procedure/Surgical Indication/ Advantages and Disadvantages	Postoperative Care/Management
Menon D; 2018 [14]	case report	1			1	21 y		right 2nd-3rd	Z-plasty + a full-thickness skin graft/neglected syndactyly, non-union of associated bones, functional impairment, aesthetic concerns. The combined approach to manage both syndactyly and non-union offers a holistic correction of the deformity, addressing both soft tissue and skeletal components and patients may face a longer recovery period due to the need for both soft tissue and skeletal healing.	
Joan Adler; 1997 [15]	case report	1	2 feet/2 webs	1		6 y		2nd-3rd digits bilaterally	rectangular flap (midline 2.0-cm incision dorsal to plantar)	Some mild maceration that occurred interdigitally was resolved via skin debridement and topical treatment with Neosporin ointment.
T. Endo; 1995 [16]	case report	1	7 webs	1		2 y		Polysyndactyly of the 1st, 2nd, and 3rd web spaces of the left foot and 1st to 4th web spaces of the right foot	simple plasties: dorsal and plantar triangular flaps and the remaining defects closed with full-thickness groin skin grafts	
D.J. Marsh; 2010 [8]	retrospective case series	15	19 conjoined toes (4 bilaterally)		1		0.3–3.2		modified Mondolfi’s technique (using interdigitating triangular skin flaps to recreate the web space and a split-thickness skin graft harvested from the instep to address the skin shortage)	
Onder Tan; 2015 [17]	retrospective case series	1	2 feet/2 webs	1		1.9 y		bilateral middle–ring	VM-plasty technique/trauma, burn contractures, congenital anomalies, oncological defects, post-surgical deformities, degenerative conditions (rheumatoid arthritis). The technique can often be performed in a single surgical stage, simplifying postoperative care, and the success of VM-plasty depends heavily on the quality and quantity of the local tissue available.	
L. De Smet; 1997 [18]	case report	2	2 feet/2 webs	1	1		0.75–9	M: third web with terminal fusion of the distal phalanges, F: for a complete syndactyly of the second web of the left foot	Flatt’s technique—the created skin defects were covered with full-thickness skin grafts	
Tristan Langlais; 2020 [19]	retrospective case series	38 4 children (4 syns) lost to follow-up	50 feet/64 webs	20	18	3.9 y	0.8–16.7	1st (*n* = 13) 2nd (*n* = 19) 3rd (*n* = 17) 4th (*n* = 15) *n* represents the number of syndactyly events	web release with a commissural dorsal flap was performed and associated with cutaneous resurfacing	Skin defects: spontaneous epithelialization, full-thickness skin graft taken from the popliteal crease, or a hyaluronic acid ester matrix
Yi-Jia Lim; 2007 [20]	retrospective case series	4	6 feet (2 syn–2 polysyn. Bilateral 2 p)	2	2	1.5 y	1.42–1.66	1 both the fourth/fifth, 1 left second/third, 1 left first to third and right first to fifth, 1 left second/third 4 cases: all patients had complete syn except 1, who had bilateral incomplete syn	planned dorsal pentagonal flap with the dorsal zig-zag incision/congenital syndactyly, polysyndactyly, aesthetic concerns, functional limitations, mild to moderate soft tissue defects/avoids donor site complications, such as scarring or infection, associated with skin grafting and mastery of the dorsal pentagonal island flap design and execution requires surgical expertise	
Tetsushi Aizawa; 2017 [21]	retrospective case series	66	88 syn toes			2.5 y	0.8–10.2	(union of the second/third toes), which accounted for 59.1%	linear skin; incisions with PSVN skin grafts led to cosmetically acceptable results without any scar contracture	
Susumu Saito; 2015 [9]	retrospective case series	8	11 webs	3	5	1/8 13 y F, 7/8 1–2 y		9 (81.8%) were corrections of simple syndactyly of the toes and 2 (18.2%) of postaxial polysyndactyly of the toes	Dorsal transversely oriented transposition flap for web reconstruction/congenital toe syndactyly, web contracture, mild to moderate tissue defects, aesthetic and functional reconstruction. The dorsal flap ensures a reliable blood supply, reducing the risk of necrosis, and accurate planning and execution of the transposition flap require experience and surgical expertise.	Prolonged use of the web space bandage contributed to the development of a very fine scar.
Kyung Rae Ko; 2020 [22]	retrospective case series	83	90 feet	41	42	27.1 y	0.6–7.8	57 feet had syndactyly affecting the fourth toe in addition to syndactyly between the duplicated toes	dorsal rectangular flap on the dorsal side of syndactyly, a rectangular flap on the plantar side was used to cover the medial side of the remaining fifth toe	
Juan Liu; 2015 [23]	retrospective case series	20	24 syn			2.5 y	1.5–8		a “plane-shaped” advancement flap (hexagonal) was used for web reconstruction without a skin graft	
Hidehiko Kawabata; 2003 [24]	retrospective case series	15	19 feet (4 cases bilateral)—30 webs: 9/30 fused completely and 21/30 fused up to the distal interphalangeal joint level	5	10	0.86 y	0.5–1.33	- 2 feet syn of the 1st and 2nd toes - 8 of the 2nd and 3rd toes - 2 of the 3rd and 4th toes - 1 foot syn 1st and 2nd, and the 3rd and 4th - both feet in one patient had syn of the 2nd, 3rd, and 4th toes four feet in two patients had syn of the 1st, 2nd, 3rd, and 4th toes and preaxial polydactyly	Open technique: the web was covered with a dorsal rectangular flap and the remaining skin defect was left open to epithelialize spontaneously (took 4 weeks) congenital syndactyly, functional Impairment, aesthetic concerns. Local flap techniques minimize complications, such as flap necrosis, and allow tension-free closure. The technique may avoid the need for extensive skin grafting in mild to moderate cases and includes wound dehiscence, infection, or contractures if the technique is not performed meticulously.	was left open to epithelialize spontaneously (took 4 weeks)
Alexander F. Mericli; 2015 [25]	retrospective case series	93	89 hand syn—32 feet syn			0.66 y	0.25–3	2nd web space	tapered M-to-V flap This approach ensures precise control of the flap dimensions to achieve a natural web contour and sufficient coverage. The technique’s effectiveness is supported by consistent outcomes over a 30-year period, making it a reliable choice and though minimized, visible scars may remain, which could be a concern for patients with keloid or hypertrophic scarring tendencies.	
Jong Ho Kim; 2016 [26]	retrospective case series	118	194 webs (155 feet), 111 nonsyndromic cases and 7 syndromic cases	52	59	1.26 y	0.5–17	80 unilateral (72.1%), the 2nd web was the most frequently involved (37.5%), 4th (30%), 1st (15%), 3rd (15%), 1st-2nd in combination (1.3%), and 2nd-3rd combination (1.3%). Among 31 bilateral cases, 2 cases were asymmetric. Among the remaining 29 symmetric bilateral cases, the 2nd web was the most frequently involved (45.2%), followed by the 1st (22.6%), and the 4th (6.5%).	dorsal triangular flap + full-thickness skin graft	
Makoto Hikosaka; 2009 [27]	retrospective case series	12	16 webs	8	4	1 (1 girl 4 year)		second webspace 12/15, third webspace 2/15, fourth webspace 2/15.	interdigital flap, rectangular and 3 triangular-shaped	The first dressing change was at one week, and subsequent dressing changes were every three to four days. After epithelialization was complete, proximal tension at the web was applied by taping

**Table 2:** Risk of bias assessment using the ROBINS-I tool.

Author and Year	Bias Due to Confounding	Bias in the Selection of Participants	Bias in the Classification of the Interventions	Bias Due to Deviations from the Intended Interventions	Bias Due to Missing Data	Bias in the Measurement of Outcomes	Bias in the Selection of the Reported Results	Overall Risk of Bias
Menon D; 2018 [14]	High	High	Low	Low	Low	Low	Low	High
Joan Adler; 1997 [15]	High	High	Low	Low	Low	Low	Low	High
T. Endo; 1995 [16]	High	High	Low	Low	Low	Low	Low	High
D.J. Marsh; 2010 [8]	High	Moderate	Low	Low	Low	Low	Low	Moderate
Onder Tan; 2015 [17]	High	High	Low	Low	Low	Low	Low	High
L. De Smet; 1997 [18]	High	High	Low	Low	Low	Low	Low	High
Tristan Langlais; 2020 [19]	Moderate	High	Low	Low	Moderate	Moderate	Low	High
Yi-Jia Lim; 2007 [20]	High	High	Low	Low	Low	Low	Low	High
Tetsushi Aizawa; 2017 [21]	High	High	Low	Low	Low	Low	Low	High
Susumu Saito; 2015 [9]	High	High	Low	Low	Low	Low	Low	High
Kyung Rae Ko; 2020 [22]	High	High	Low	Low	Low	Low	Low	High
Juan Liu; 2015 [23]	High	High	Low	Low	Low	Low	Low	High
Hidehiko Kawabata; 2003 [24]	High	High	Low	Low	Low	Low	Low	High
Alexander F. Mericli; 2015 [25]	High	High	Low	Low	Low	Low	Low	High
Jong Ho Kim; 2016 [26]	High	High	Low	Low	Low	Low	Low	High
Makoto Hikosaka; 2009 [27]	High	High	Low	Low	Low	Low	Low	High

**Table 3:** Complications of surgical procedures.

Author and Year	Complications
Menon D; 2018 [14]	none
Joan Adler; 1997 [15]	some mild maceration occurred interdigitally
T. Endo; 1995 [16]	7 keloids
D.J. Marsh; 2010 [8]	1 with mild web creep; 1 with skin graft failure that healed by a secondary intervention; 1 with hypertrophic scarring
Onder Tan; 2015 [17]	none
L. De Smet; 1997 [18]	1 with keloid; 1 with web creep and keloid
Tristan Langlais; 2020 [19]	28.1% recurrence; 6 with keloids (9.4%); 1 with scar retraction (1.6%)
Yi-Jia Lim; 2007 [20]	1 with partial synechiae; 1 with keloid
Tetsushi Aizawa; 2017 [21]	1 with hyperpigmentation 1; 3 with mild axis displacement
Susumu Saito; 2015 [9]	
Kyung Rae Ko; 2020 [22]	17 with delayed healing or early postoperative wound infections; among these, 7 patients showed postoperative thickening or advancement of the web
Juan Liu; 2015 [23]	1 with keloid
Hidehiko Kawabata; 2003 [24]	skin defect
Alexander F. Mericli; 2015 [25]	1 with foot and toe discomfort while ambulating, web creep (12 web spaces; 9%)
Jong Ho Kim, 2016 [26]	1.6% with postoperative complications: 1 needed secondary surgery to correct web creep (0.52%), 1 with hypertrophic scarring (0.52%), 1 with a pressure sore of the heel (0.52%) that resolved with conservative management
Makoto Hikosaka; 2009 [27]	none

## Data Availability

The data presented in this study are available upon request from the corresponding author.

## Supplementary Materials

The following supporting information can be downloaded at: https://www.mdpi.com/article/10.3390/jcm14030830/s1, PRISMA 2020 Checklist [34].
